# Dose error from deviation of dwell time and source position for high dose-rate ^192^Ir in remote afterloading system

**DOI:** 10.1093/jrr/rru001

**Published:** 2014-02-23

**Authors:** Hiroyuki Okamoto, Ako Aikawa, Akihisa Wakita, Kotaro Yoshio, Naoya Murakami, Satoshi Nakamura, Minoru Hamada, Yoshihisa Abe, Jun Itami

**Affiliations:** Department of Radiation Oncology, National Cancer Center Hospital, 104-0045, Tokyo, Japan

**Keywords:** dwell time, source position, ^192^Ir, dose error, RALS

## Abstract

The influence of deviations in dwell times and source positions for ^192^Ir HDR-RALS was investigated. The potential dose errors for various kinds of brachytherapy procedures were evaluated. The deviations of dwell time Δ*T* of a ^192^Ir HDR source for the various dwell times were measured with a well-type ionization chamber. The deviations of source position Δ*P* were measured with two methods. One is to measure actual source position using a check ruler device. The other is to analyze peak distances from radiographic film irradiated with 20 mm gap between the dwell positions. The composite dose errors were calculated using Gaussian distribution with Δ*T* and Δ*P* as 1σ of the measurements. Dose errors depend on dwell time and distance from the point of interest to the dwell position. To evaluate the dose error in clinical practice, dwell times and point of interest distances were obtained from actual treatment plans involving cylinder, tandem-ovoid, tandem-ovoid with interstitial needles, multiple interstitial needles, and surface-mold applicators. The Δ*T* and Δ*P* were 32 ms (maximum for various dwell times) and 0.12 mm (ruler), 0.11 mm (radiographic film). The multiple interstitial needles represent the highest dose error of 2%, while the others represent less than approximately 1%. Potential dose error due to dwell time and source position deviation can depend on kinds of brachytherapy techniques. In all cases, the multiple interstitial needles is most susceptible.

## INTRODUCTION

The radioactive source and seed used in brachytherapy can emit photons or electrons in all directions. Dosimetrically, this means that the dose gradient around a brachytherapy source is very steep, and the dose decreases exponentially with the thickness of the material or tissue. Therefore, by implanting the source near or into the tumor region, high conformity of prescribed dose to the target and reduction of unwanted dose to healthy organs surrounding the tumor region can be obtained.

The use of CT or MR images in treatment planning for brachytherapy has been widely employed as image-guided brachytherapy (IGBT). Over the past few years, a number of studies have been conducted on various IGBT techniques [1–4]. Dose distributions can be customized to fit the target for each patient by referring to actual anatomy from CT or MR images instead of film-based planning. Furthermore, recently, sophisticated dwell time optimization techniques, such as graphical optimization and inverse planning anatomy-based dose optimization, has been implemented in commercial treatment planning systems (TPSs) [5–9].

Treatment parameters (including dwell times and dwell positions of the radioactive source calculated by TPSs) are transferred to the treatment machine, such as a remote afterloading system (RALS), manufactured e.g. by Nucletron, an Elekta company (MicroSelectron^®^, Stockholm, Sweden), Varian Medical Systems Inc. (VariSource^TM^, Palo Alto, CA, USA), or Eckert & Ziegler Bebig SA (MultiSource^®^, Seneffe, Belgium). The RALS should provide high precision control of the movement of the radioactive source as planned in terms of the dwell times and dwell positions in the applicators in which the source moves. Mechanical accuracies of movement of the radioactive source have been referred to in some quality assurance/quality control (QA/QC) guidelines for high-dose-rate (HDR) RALS [10–13]. For example, AAPM Task Group 40 and Task Group 56 refer to a tolerance of source positioning of ±1 mm [10, 11]. ESTRO Booklet No. 8 recommend an action level of source positioning accuracy of ±2 mm [12]. The tolerance or action level can be applicable for the scheduled QA/QC treatment machine. However, it is not obvious how these figures relate to clinical practice, and they lack practical value in terms of clinical influence. It has been further suggested that these values can be applied to all of the irradiation techniques in brachytherapy, such as surface-mold and interstitial brachytherapy, etc. Actually, dose error due to deviation of the source position depends on the distance from the source according to the inverse square law. A large dose error can be observed near the source, but the dose error far from the source is less.

Similarly, the impact of dose change caused by dwell time error can be expected to be linearly dependent on total dwell time. For instance, a tiny dwell time error can have a large impact in the case of low dwell time. In particular, the graphical optimization and inverse-planning anatomy-based dose optimization of the commercially available TPSs performs calculations of the dwell times without putting a lower limit on dwell time. Therefore, it is important to investigate dose error for such brachytherapy techniques, which freely use a low dwell time.

Dose error from mechanical uncertainties of movement of the source can depend on the kind of irradiation technique used, and its clinical influence is not fully understood. Our purpose in this study was to evaluate the dose error for clinical treatment plans involving a range of irradiation applicators/catheters, such as cylinder, tandem/ovoid, tandem/ovoid with a small number of interstitial needle applicators (‘Combination-brachytherapy’), multiple interstitial needle applicators, and surface-mold applicators for scalp tumors.

In this study, deviations in dwell time and source position were measured for a number of techniques. Finally, the influence of the dose error due to both deviations was investigated for actual treatment plans involving the various applicators or catheters listed above.

## MATERIALS AND METHODS

### Measurements of deviation of dwell time and source position

For ^192^Ir HDR-RALS (MicroSelectron^®^ V2, Nucletron, an Elekta company), the deviation in dwell time *ΔT* and source position *ΔP* were measured for a range of techniques [12–16]. For two measurements, the air kerma strength was ∼30.0 mGy m^2^ h^−1^. The Nucletron RALS has a nominal time resolution of 0.1 s. As shown in Eq. (1), dwell time, *T*_*m*_ was obtained from the amount of electric charge measured by a well-type ionization chamber (WIC) and an electrometer (HDR-1000 plus and MAX-4000, Standard Imaging Inc., Middleton, WI, USA). The correction factor *k*_*TP*_ was applied to convert the cavity air mass at the reference conditions (temperature *T*_0_ 22°C and pressure *P*_0_ 101.3 kPa) [13].

Dwell time *T*_*m*_ can be obtained from the amount of net electric charge (*M − M*_*EF*_) and the current *A*_0_ of the WIC during the irradiation, as shown in the following equation.

(1) $$T_{m}=\frac{M-M_{EF}}{A_{0}}$$

*M*_*EF*_ means amount of electric charge in the end effect of a source, and it can be regarded as a constant independent of dwell time. Therefore, a standard deviation of dwell time *T*_*m*_ can be equal to that of *M*/*A*_0_. The dwell time deviation *ΔT* was defined as a maximum value for a standard deviation of 10 measurements of *M*/*A*_0_ for nominal dwell time *T* of 0.1, 0.2, 0.5, 1.0, 5.0, 15.0 and 30.0 s.

**Fig. 1.**
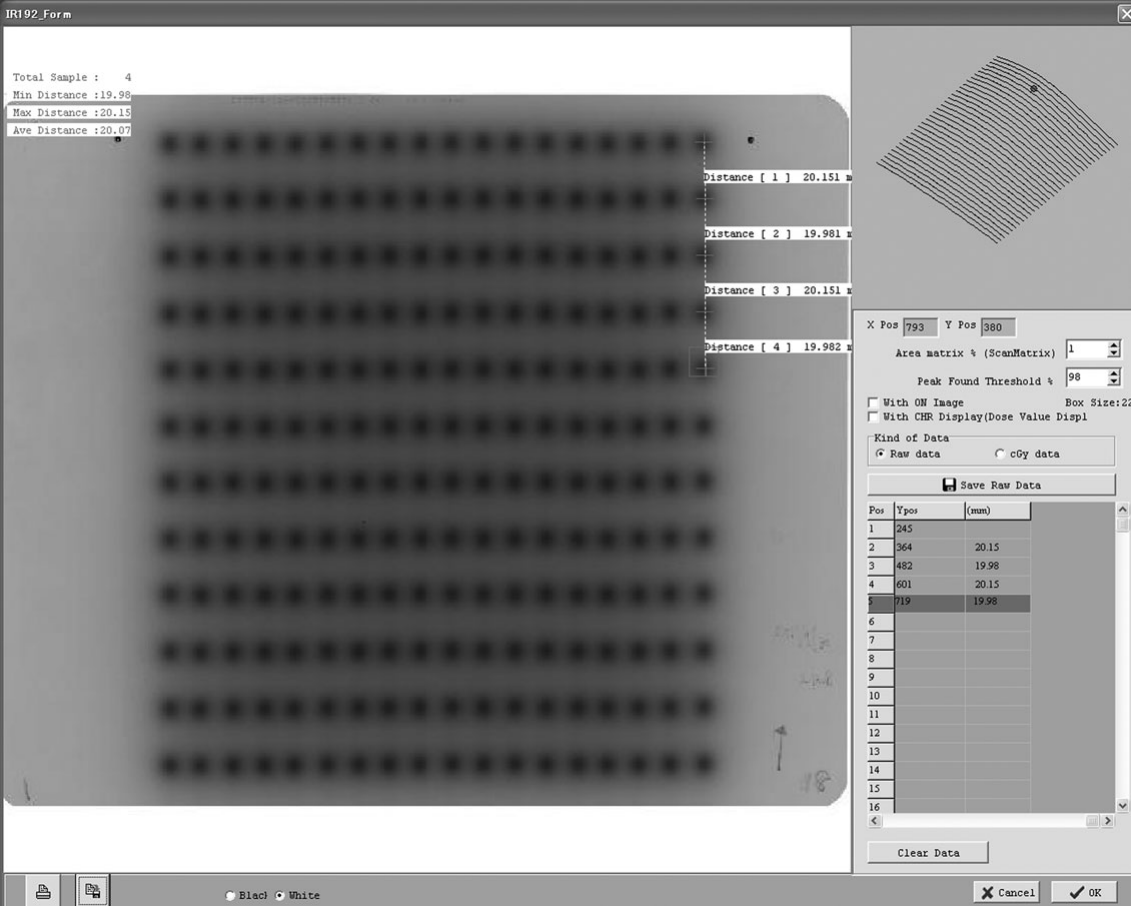
Radiographic film EDR2 (Carestream Health Inc.) was analyzed for the measurement of source position accuracy using film QA software DD-system Ver. 10.21 (R-TECH Company).

The deviations in the source positions were measured with two methods. One method involved measuring the position of the source with a source position check ruler device specifically developed for MicroSelectron^®^ and provided by Nucletron, an Elekta company. The MicroSelectron^®^ was set up to place the source in a certain position via the ruler connected by a straight transfer tube. By reading the actual source position, the standard deviation of the position deviation was obtained. A total of 11 measurements were performed to obtain a standard deviation. The other method involved irradiation of radiographic film (EDR2, Carestream Health Inc., Rochester, NY, USA) with applicators tightly attached to it. Irradiation was performed with 2-cm gaps between the dwell positions, and the number of gaps was 198. The exposed films were digitized with an EPSON ES-8500 flatbed scanner under resolution conditions of 600 dpi (equivalent to 0.042 mm/pixel) and a 16-bit gray scale. The digitized images were analyzed using film QA software (DD-system Ver. 10.21, R-TECH Company, Tokyo, Japan) as shown in Fig. 1. The source position deviation *ΔP* was defined as the maximum of a standard deviation for two tests (ruler and radiographic film).

### Evaluation of dose error in single catheter method

**Fig. 2.**
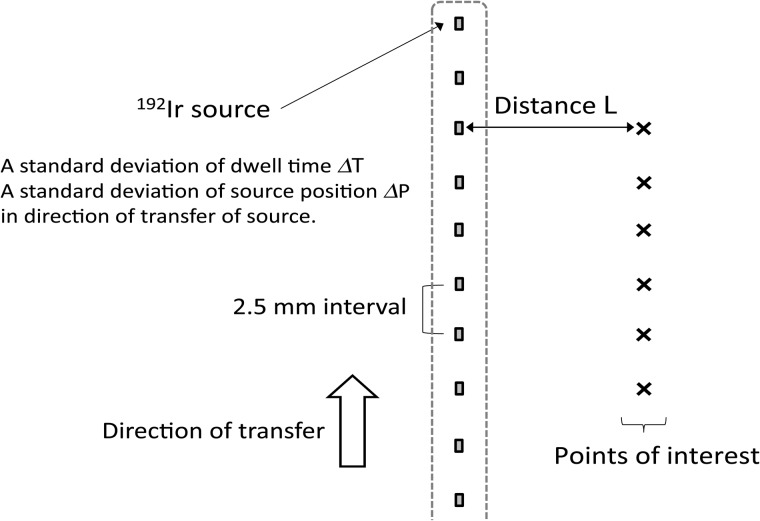
Dose errors at points of interest for a single catheter calculated according to the AAPM TG-43 protocol [17–19] due to the two deviations: dwell time deviation Δ*T* and source position deviation Δ*P*.

As shown in Fig. 2, we calculated the dose errors at the points of interest due to deviations in both dwell time *ΔT* and source position *ΔP*, using the methods as described in the previous section. Two deviations were used as a standard deviation (according to Gaussian distribution) in the development environment of Microsoft Visual Studio 2010 instead of the commercial TPS. Dose calculation with a grid size of 1.0 mm was performed according to AAPM TG-43 protocol [17–19], as is typically used for dose calculation in brachytherapy TPS. Points of interest were placed at a distance *L* from each dwell position in the perpendicular direction of a catheter. A total of 10 dwell positions with a 2.5-mm distance from each source was investigated. Dose deviations were defined as the mean value for a standard deviation of the dose at the points of interest in 10 trials.

### Evaluation of dose calculation error in various clinical treatment plans

Dose error at the points of interest is affected by two factors: dwell time and distance of the point of interest from the source. In order to evaluate the influence of the two deviations, *ΔT* and *ΔP*, for a clinical treatment plan, we measured the dwell times and distance to points of interest in the actual treatment plan for a range of applicator types, such as cylinder, tandem/ovoid, combination-brachytherapy, multiple interstitial needle applicators, and surface-mold applicators for scalp tumors, in TPS Oncentra Brachy Ver. 4.1.0.132 (Nucletron, Veenendaal, Netherland). Table 1 provides information about the treatment plans for the various applicator types investigated here.

**Table 1.** Treatment plans using various kinds of applicators or catheters

Applicators or catheters	# treatment plans	Prescribed dose (Gy)	Method of optimizing dose distribution
Cylinder	16	6	Dose point optimization to 5 mm under vaginal surface
Tandem/ovoid	17	6	Manchester system
Combination-brachytherapy	9	6	Inverse planning based on dose–volume histogram and graphical optimization
Multiple interstitial needle applicators	8	6	
Surface-mold for scalp tumor	5	2–2.5	Dose point and graphical optimization

As time passes, dwell times for the same treatment plan must be increased in order to compensate for the decay of the radioactivity source (half-life of ^192^Ir = 73.83 d). In other words, air kerma strength is higher, e.g., just after the replacement of an ^192^Ir source. A maximum value *S*_*K*_ of 48.56 mGy m^2^ h^−1^ was obtained from the 43 measurements taken between February 2002 and December 2012 in our institute (average ± 1SD, 42.21 ± 3.06 mGy m^2^ h^−1^). Dwell times for all of the treatment plans listed in Table 1 were recalculated using the maximum *S*_*K*_ of 48.56 mGy m^2^ h^−1^, because a large dose error can be observed in the case of low dwell time.

In this study, the distance to the point of interest was defined by the equation below. We used the geometrical averaged distance *r*_*g*_ of the point of interest from catheter, *r*_*g*_.

(2) $$r_{g}=\sqrt{V_{100\%}/\sum_{i}\pi l_{i}}$$

This equation can be derived from $\sum_{i}\pi r_{g}^{2}\times l_{i}=V_{100\%}$. *V*_100%_ means the irradiated volume receiving the prescribed dose or more, and it can be obtained from the dose–volume histogram in the TPS. The *l*_*i*_ is assumed to be the length of treatment region of the *i*th catheter. The gap between the dwell positions was set to a 2.5-mm interval. Therefore, the length of treatment region *l*_*i*_ could be expressed as a multiplication of the 2.5 mm interval by the number of dwell positions.

## RESULTS

### Measurements of deviation of dwell time and source position

Dwell time deviation was measured with a WIC. The standard deviations of dwell time for 10 measurements are shown in Table 2. From these results, the maximum standard deviation was found to be 32 ms for a dwell time of 30.0 s.

**Table 2.** Dwell time deviation for Nucletron RALS measured with a WIC

Dwell time (s)	0.1	0.2	0.5	1.0	5.0	15.0	30.0
Deviation (ms)	18	17	23	23	29	23	32

Deviation of the source position in the Nucletron RALS was measured using the two methods. The standard deviation of the source position was 0.12 mm and 0.11 mm in measurements using either the source position check ruler device (*n* = 11) or radiographic film (*n* = 198), respectively. The dwell time and source position deviation in this study were regarded as a standard deviation according to Gaussian distribution, Δ*T* = 32 ms and Δ*P* = 0.12 mm (worst case scenario).

### Dose error in a single catheter by calculation

**Fig. 3.**
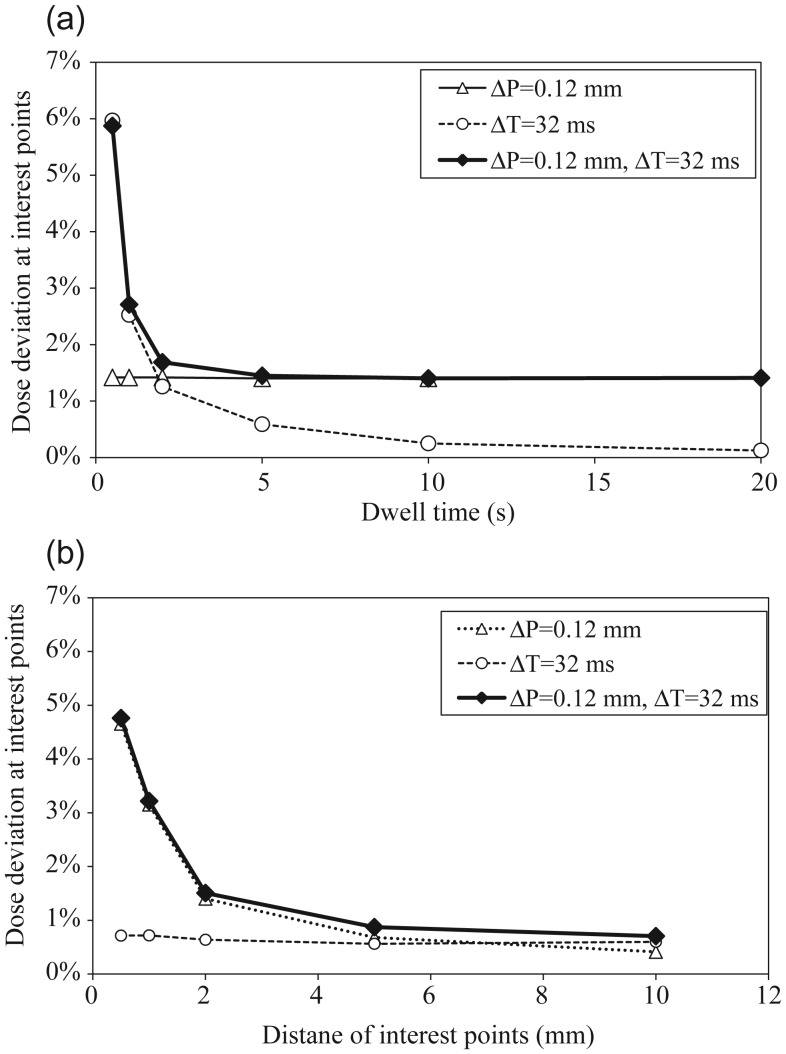
Dose error for Δ*T* = 32 ms and Δ*P* = 0.12 mm: (**a**) dose error at points of interest placed at a fixed distance of 5 mm as a function of dwell time by calculation. (**b**) Dose error for a fixed dwell time of 2 s as a function of distance to points of interest by calculation.

Figure 3a depicts the calculated dose errors as a function of dwell time with points of interest placed at a fixed distance of 5 mm with the measured two deviations, using Δ*T* = 32 ms and Δ*P* = 0.12 mm as the standard deviation according to the Gaussian distribution. Dose errors were calculated with three conditions: with either *ΔT*, *ΔP,* or both of them to reveal the contribution of each deviation to the total dose error. Using only *ΔP*, the dose error was constant independent of the dwell time. Using only *ΔT*, the dose error decreased with an increased dwell time. For both two deviations, the dose error also decreased with an increase in dwell time, and was a composite of the graphs when using only Δ*P* and only Δ*T*. Figure 3b depicts the calculated dose errors as a function of distance *L* to the point of interest with a fixed dwell time of 2 s and the two measured deviations, Δ*T* = 32 ms and Δ*P* = 0.12 mm generating the standard deviation according to Gaussian distribution. Dose errors were also calculated according to these assumptions in Fig. 3a. With only *ΔP*, the dose error decreased with an increase in distance *L* to the point of interest. The dose error approached zero, because *ΔP* is less influential for a point distant from the source. Using only *ΔT*, the dose error remained constant, because dose was linearly proportional to the dwell time. With both deviations, the dose error also decreased with increase in distance to the point of interest, and was a composite of the graphs with only Δ*P* and only Δ*T*.

**Fig. 4.**
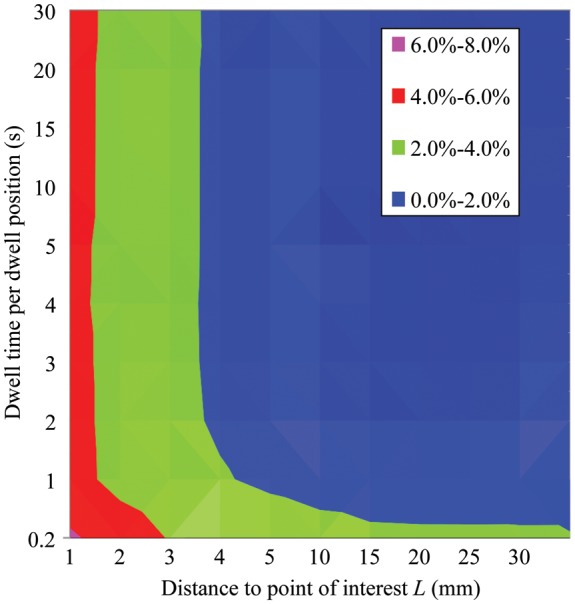
2D-plot of dose error for dwell times and distance to point of interest *L* for Δ*T* = 32 ms and Δ*P* = 0.12 mm.

As shown in Fig. 3, the dose error depended on two factors: dwell time and distance to the point of interest. Figure 4 depicts a 2D-plot of the dose errors with the two measured deviations, Δ*T* = 32 ms and Δ*P* = 0.12 mm. As shown in the figure, a high dose error could be observed near the source for a small dwell time.

### Dwell time and distance to the point of interest for clinical treatment plans

**Fig. 5.**
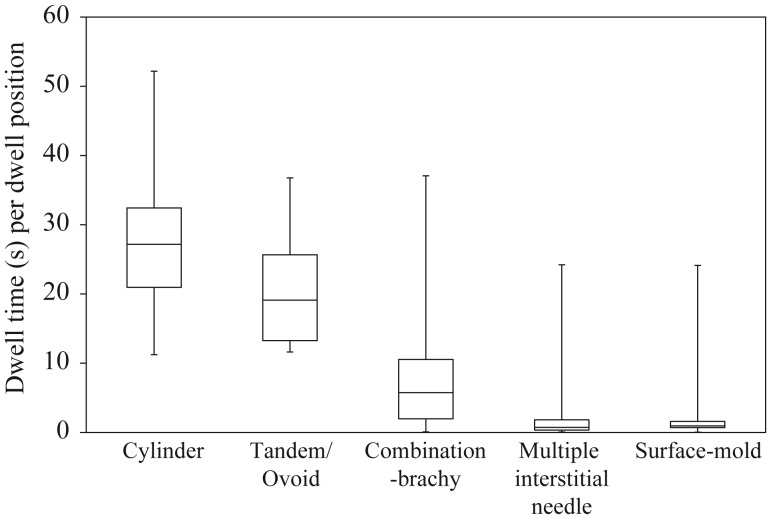
The box-and-whisker plot of the dwell times per source position for the clinical treatment plans (see Table 1).

**Fig. 6.**
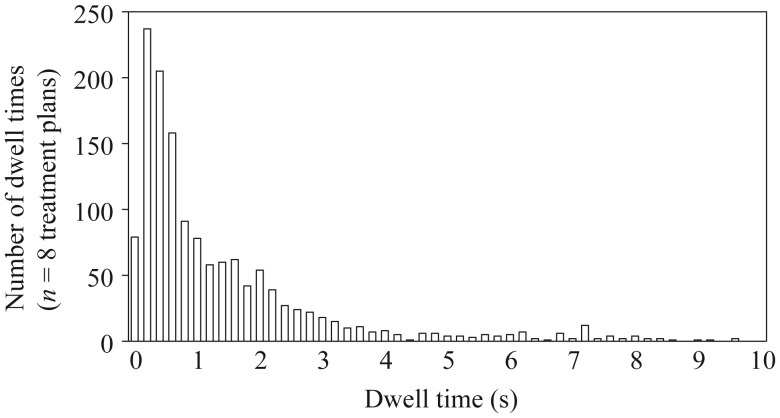
Distribution of dwell times for the multiple interstitial needle applicators (*n* = 8 treatment plans). Inverse planning and graphical optimization are used to optimize dwell time in the Treatment Planning System.

Figure 5 depicts the box-and-whisker plot of dwell times per dwell position in the actual clinical treatment plans as described in Table 1. The median dwell time for each dwell position was 27.18, 19.10, 5.74, 0.73 and 0.95 s for the cylinder, tandem/ovoid, combination-brachytherapy, multiple interstitial needle applicators, and surface-mold applicators for scalp tumors, respectively. The lower quartile of dwell times was 20.96, 13.26, 1.97, 0.34 and 0.69 s, respectively. For instance, Fig. 6 depicts a distribution of dwell times for the multiple interstitial needle applicators (*n* = 8 treatment plans).

**Fig. 7.**
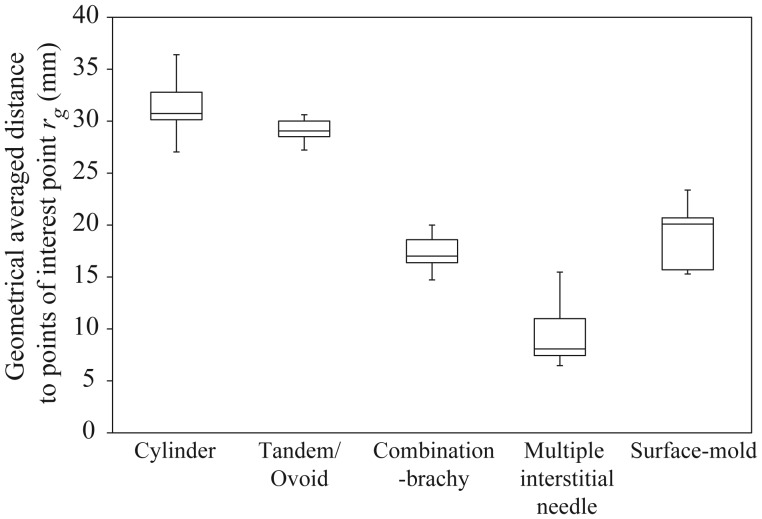
The box-and-whisker plot of the geometrical averaged distance to the point of interest derived from Eq. (2) for clinical treatment plans (see Table 1).

Figure 7 depicts the box-and-whisker plot of the geometrical averaged distance to the point of interest *r*_*g*_ derived from Eq. (2) for the clinical treatment plans (see Table 1). The median value of *r*_*g*_ was 30.73, 29.06, 17.01, 8.08 and 20.10 mm in the cylinder, tandem/ovoid, combination-brachytherapy, multiple interstitial needle applicators, and surface-mold applicators for scalp tumors, respectively. The lower quartile of *r*_*g*_ was 30.14, 28.51, 16.37, 7.44 and 15.70 mm, respectively. Furthermore, minimum *r*_*g*_ was 27.03, 27.22, 14.72, 6.47, and 15.29 mm, respectively.

### Potential dose error for clinical treatment plans

We adopted the results of the dose error for a single catheter by calculations from clinical treatment plans. We introduced the median value of Figs 5 and 7 as a representative expression of the treatment plans. This condition assumed that the dwell time and the distance to the point of interest for all activations can be considered equivalent to the median value. Table 3 lists the potential dose errors that were obtained from the 2D-plot of the dose error indicated by the two median values.

**Table 3.** Potential dose errors for clinical treatment plans (see Table 1) with Δ*T* = 32 ms and Δ*P* = 0.12 mm from measurements

Applicators or catheters	(Δ*T*, Δ*P*) = (32 ms, 0.12 mm)
	Potential dose error
Cylinder	∼0.1%
Tandem/ovoid	∼0.2%
Combination-brachytherapy	∼0.3%
Multiple interstitial needle applicators	∼2.0%
Surface-mold for scalp tumor	∼1.3%

## DISCUSSION

In brachytherapy, there are many uncertainties in the prescribed dose for the overall treatment process such as the uncertainty in calculating the absolute dose [17–19] and the effect of inhomogeneity [20–24], the mechanical accuracy of the source movement, and the intra- and inter-fractional displacements of the applicators or catheters, which cause substantial dose deviation from the planned dose. Of these uncertainties, we focus upon the dose errors caused only by the mechanical uncertainties of movement of the source in ^192^Ir HDR-RALS in terms of dwell time and source position.

The dwell time deviation was obtained from the amount of electric charge from the WIC during the irradiation [see Eq. (1)]. This measurement is considered to be influenced by the deviation, both in dwell time and source position. However, we could neglect the influence of deviation in the source position, for the following reason. For this measurement, the ^192^Ir source was located at a predetermined position in the WIC where the amount of electric charge represented the maximum. The behavior of the amount of electric charge in this region can be comparatively flat for a variable source position. For example, a displacement of 0.1 mm in the source position causes a change in the amount of electric charge by 0.001%. This corresponds to a dwell time of 1 ms. Therefore, the amount of electric charge is weakly dependent on the deviation of the source position.

The Nucletron RALS has high precision control of the dwell time of a source independent of dwell time (Table 2). We determined source position deviation via two methods, and the results obtained from each in this study were similar. Mechanical accuracy of the source movement in the short term was also evaluated. Actually, deterioration of the motor controlling source movement can happen abruptly or can gradually worsen in the long term. The long-term mechanical accuracy needs to be further investigated.

From Fig. 3, it is clear how dose error at the point of interest for a single catheter can change with variable dwell time and distance to the point of interest from a source. In the case of low dwell time and placement of the point of interest near a source, dose error can be expected to be high. Therefore, it is important that the effect of both deviations is taken into account when evaluating potential dose error for different the brachytherapy techniques. Of all the brachytherapy techniques investigated, the smallest and largest dwell times were obtained with the multiple interstitial needle and cylinder applicators, respectively (see Fig. 5). Additionally, the brachytherapy techniques having the smallest and largest distance to the point of interest from the source were also the multiple interstitial needle and cylinder applicators, respectively (see Fig. 7).

As shown in Table 3, all of the catheters or applicators represented fell within a 2% dose error. This is because the Nucletron RALS has high precision control of the movement of the radioactive ^192^Ir source, Δ*T* = 32 ms and Δ*P* = 0.12 mm. The finding is that the potential dose error depends on the kind of brachytherapy technique used. Of all the techniques investigated, the multiple interstitial needle applicators could introduce the most error, ∼2.0% potential dose error. A procedure for evaluation of the potential dose error, applicable for all brachytherapy techniques, was proposed in this study. The authors recommended that the user establish the 2D-plot for dwell time and distance to the point of interest with the measured accuracy of movement of the source, and need to evaluate the potential dose error caused by mechanical uncertainties of movement of the source for the brachytherapy techniques used in a particular institute.

## CONCLUSIONS

Mechanical uncertainties in the ^192^Ir HDR-RALS (Nucletron, an Elekta company), a dwell time deviation Δ*T* of 32 ms and a source position deviation Δ*P* of 0.12 mm were obtained from measurements. The finding was that the Nucletron HDR-RALS has high precision control of the movement of the radioactive ^192^Ir source in terms of dwell time and source position. The potential dose error caused by the two deviations depends on the kind of brachytherapy technique used. Of the techniques studied, multiple interstitial needle applicators have the highest susceptibility to dwell time deviation and source position deviation.

## FUNDING

This study was partially supported by the Cancer Research Development Fund (23-A-13) of the National Cancer Center and the Cancer Clinical Research Fund of the Ministry of Welfare, Health and Labor.
